# The needs of families of pediatric cancer survivors: challenges and developments in psychosocial support services

**DOI:** 10.1038/s41390-024-03662-x

**Published:** 2024-10-22

**Authors:** Verena Paul, Laura Inhestern, Désirée Sigmund, Jana Winzig, Stefan Rutkowski, Gabriele Escherich, Corinna Bergelt

**Affiliations:** 1 Department of Medical Psychology, University Medical Center Hamburg-Eppendorf, Hamburg, Germany; 2 German Center for Child and Adolescent Health (DZKJ), partner site Hamburg, Hamburg, Germany; 3 Department of Pediatric Hematology and Oncology, University Medical Center Hamburg-Eppendorf, Hamburg, Germany; 4 Department of Medical Psychology, University Medicine Greifswald, Greifswald, Germany; 5 German Center for Child and Adolescent Health, partner site Rostock/Greifswald, Greifswald, Germany

## Abstract

- Caru et al. emphasize the positive of physical activity during and after treatment for children with cancer, highlighting its significance for improving health outcomes.
- In Germany, exercise therapy has not yet been integrated into standard post-treatment care; however, efforts are underway to establish a nationwide framework that enhances these services.
- Given the complexity of the challenges faced by families, a family-centered approach to psychosocial support services is essential for effectively addressing their multifaceted needs.

I would like to express our gratitude to Caru and Levesque for their thoughtful commentary^1^ on our recent publication “Addressing gaps and enhancing experiences in support services for families of pediatric cancer survivors” in Pediatric Research.^2^ Their insights illuminate critical aspects of pediatric oncology care and initiate an important dialogue surrounding the needs of both survivors and their families. In our study, we emphasized the necessity of comprehensive support systems that address the multifaceted requirements of families affected by childhood cancer after primary treatment.^2^ Caru et al. rightly highlight the importance of physical activity during and after primary treatment of childhood cancer.^1^ Research consistently demonstrates that appropriate levels of physical activity can significantly reduce late effects associated with cancer and cancer treatments, thereby promoting better health outcomes for survivors.^3,4^ The growing importance of physical activity during and after cancer treatment cannot be overstated. The lack of explicit mention of exercise therapy by parents could be attributed to several factors, which we will briefly explain below.

In Germany, physiotherapy has long been integrated as a standard component of care for pediatric cancer patients, both during and after primary therapy. Access to physiotherapy in Germany is relatively straightforward, with widespread offerings typically covered by health insurance when needed. In contrast, exercise therapy has not yet been integrated into standard care for cancer survivors.^5^ Many projects currently rely on third-party funding, which limits their integration into consistent healthcare practices and makes them unavailable nationwide. However, it is encouraging to see increasing efforts aimed at establishing networks and implementing long-term strategies, which seek to create a more uniform approach to integrating physical activity into pediatric oncology care.^5^ The 2021 released AWMF-S2k guideline explicitly state that movement-promoting and movement-therapeutic support should be tailored to the individual needs of children and adolescents, taking into account their life circumstances, motivation, and any health limitations.^6^ However, the exercise therapy program is still under development and not evenly accessible to all patients.

Since we did not specifically inquire about individual support services in the interviews, it is also possible that parents mistakenly used the commonly known term ‘physiotherapy’ synonymously with ‘exercise therapy’. It is important to note that exercise therapy is often provided and utilized within the context of other programs, such as inpatient rehabilitation. Thus, participants may not regard exercise therapy as a distinct category. As the interview guideline did not list support services individually; parents might have summarized them under the term ‘rehabilitation’ or ‘physiotherapy’. We concur with Caru et al. that there is an information gap resulting from this issue.^1^ A detailed enumeration of these services would indeed be interesting and worthy of further investigation; however, it would have exceeded the scope of the publication mentioned.

In our manuscript “Addressing gaps and enhancing experiences in support services for families of pediatric cancer survivors“, we further highlighted that, despite high demand, psychosocial support services are utilized to a limited extent.^2^ Caru et al. specifically pointed out the importance of psychoeducation and family-oriented services,^1^ which we fully support. In Germany, an increasing number of family-orientated programs are being developed and evaluated. Nevertheless, it is crucial to facilitate access and to continue raising awareness of the needs of all family members. Furthermore, Caru et al. emphasize on the connection between caregiver burden and emotional distress is a vital point, that warrants further exploration in future research.^1^ It is imperative that we work toward creating an integrative healthcare environment that addresses the needs of all family members. We appreciate Caru’s and Levesques’s valuable input on these matters and are dedicated to continuing our engagement with this topic. Enhancing the support infrastructure for families of pediatric cancer survivors remains a crucial objective, and their insights contribute significantly to this ongoing dialogue.
